# Gastrotricha: A Marine Sister for a Freshwater Puzzle

**DOI:** 10.1371/journal.pone.0031740

**Published:** 2012-02-14

**Authors:** M. Antonio Todaro, Matteo Dal Zotto, Ulf Jondelius, Rick Hochberg, William D. Hummon, Tobias Kånneby, Carlos E. F. Rocha

**Affiliations:** 1 Department of Biology, University of Modena and Reggio Emilia, Modena, Italy; 2 Department of Invertebrate Zoology, Swedish Museum of Natural History, Stockholm, Sweden; 3 Department of Biological Sciences, University of Massachusetts Lowell, Lowell, Massachusetts, United States of America; 4 Department of Biological Sciences, Ohio University, Athens, Ohio, United States of America; 5 Departamento de Zoologia, Instituto de Biociências, Universidade de São Paulo, São Paulo, Brazil; University of Veterinary Medicine Hanover, Germany

## Abstract

**Background:**

Within an evolutionary framework of Gastrotricha *Marinellina flagellata* and *Redudasys fornerise* bear special interest, as they are the only Macrodasyida that inhabit freshwater ecosystems. Notwithstanding, these rare animals are poorly known; found only once (Austria and Brazil), they are currently systematised as *incertae sedis*. Here we report on the rediscovery of *Redudasys fornerise*, provide an account on morphological novelties and present a hypothesis on its phylogenetic relationship based on molecular data.

**Methodology/Principal Findings:**

Specimens were surveyed using DIC microscopy and SEM, and used to obtain the 18 S rRNA gene sequence; molecular data was analyzed cladistically in conjunction with data from 42 additional species belonging to the near complete Macrodasyida taxonomic spectrum. Morphological analysis, while providing new information on taxonomically relevant traits (adhesive tubes, protonephridia and sensorial bristles), failed to detect elements of the male system, thus stressing the parthenogenetic nature of the Brazilian species. Phylogenetic analysis, carried out with ML, MP and Bayesian approaches, yielded topologies with strong nodal support and highly congruent with each other. Among the supported groups is the previously undocumented clade showing the alliance between *Redudasys fornerise* and *Dactylopodola agadasys;* other strongly sustained clades include the densely sampled families Thaumastodermatidae and Turbanellidae and most genera.

**Conclusions/Significance:**

A reconsideration of the morphological traits of *Dactylopodola agadasys* in light of the new information on *Redudasys fornerise* makes the alliance between these two taxa very likely. As a result, we create *Anandrodasys* gen. nov. to contain members of the previously described *D. agadasys* and erect Redudasyidae fam. nov. to reflect this novel relationship between *Anandrodasys* and *Redudasys*. From an ecological perspective, the derived position of *Redudasys*, which is deeply nested within the Macrodasyida clade, unequivocally demonstrates that invasion of freshwater by gastrotrichs has taken place at least twice, in contrast with the single event hypothesis recently put forward.

## Introduction

The cosmopolitan phylum Gastrotricha includes approximately 760 microscopic, aquatic species divided into two orders: Macrodasyida, and Chaetonotida. Macrodasyidans are elongate, vermiform animals counting about 280 species; as a rule they are hermaphroditic and inhabit sands of the marine environment. However, with regard to the environment, two notable exceptions exist: *Marinellina flagellata* Ruttner-Kolisko, 1955 and *Redudasys fornerise* Kisielewski, 1987. In fact, both species have been reported from freshwater habitats: an Austrian, alpine stream and a Brazilian, artificial reservoir, respectively [1], [2]. The two species have been found only once and, due to the scanty nature of the original descriptions (especially true for *M. flagellata*), their phylogenetic alliances appear uncertain; as a consequence, *Marinellina* and *Redudasys* are currently systematised as *incertae sedis* [3]. We trust that surveys of new material, especially using modern methodologies of investigation, will provide new information that will clarify the taxonomic status of these enigmatic animals and hopefully explain the invasion of freshwater ecosystems by an originally marine taxon.

Attempts to rediscover the European animals has in part failed, i.e., research in the type locality (i.e. river) has yielded no results (W.D. Hummon, unpublished) but a macrodasyidan gastrotrich has been found in another Austrian stream (J.M. Schmidt-Araya, personal communication). Based on the pictures of this animal, it may however be arguable to identify it as *Marinellina flagellata* [4].

Here we report on the rediscovery of *R. fornerise* in the Brazilian type locality. Beside a morphological account based on light (DIC) and electron microscopy (SEM) of this species, we provide results of three phylogenetic analyses based on 18 S rRNA gene sequence data from the Brazilian animals and from other species belonging to the near complete Macrodasyida taxonomic spectrum.

## Materials and Methods

**Figure 1:**
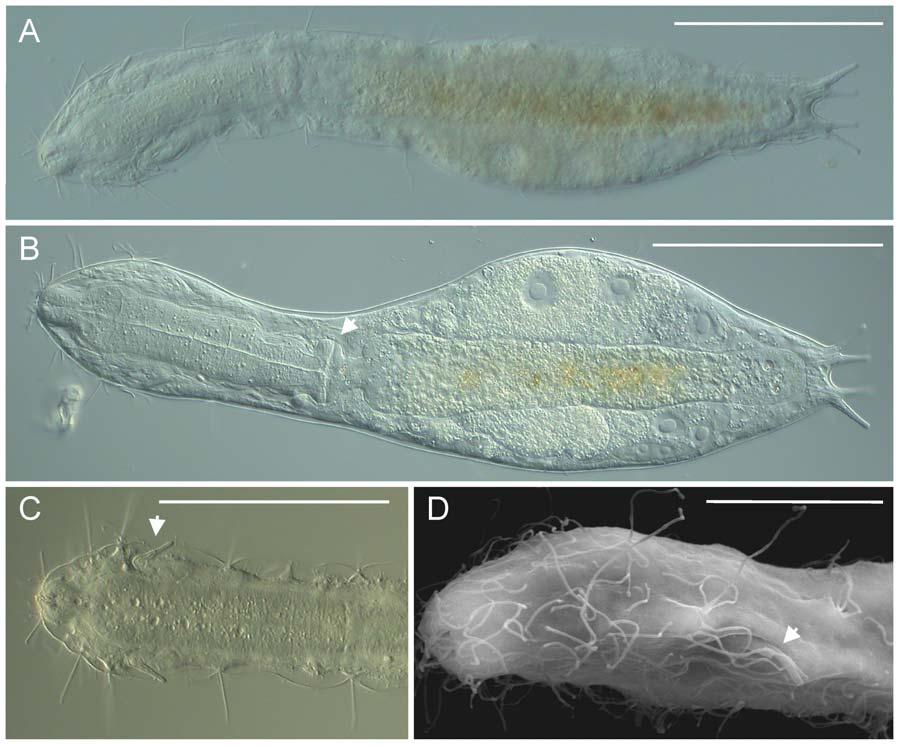
*Redudasys fornerise*. A, habitus of a fully relaxed adult specimen; B, habitus at different focal plane of a different specimen slightly contracted and compressed, arrowhead indicates the pharyngeal pores; C, anterior end of a third specimen showing the insertion of the anterior adhesive tubes (arrowhead); D, close-up of the anterior end of a fourth specimen, showing the arrangement of sensorial cilia and the insertion of the anterior adhesive tubes (arrowhead). A–C, DIC photomicrographs, D, SEM photomicrographs. Scale bars, A–C, 100 µm, D, 20 µm.

**Figure 2:**
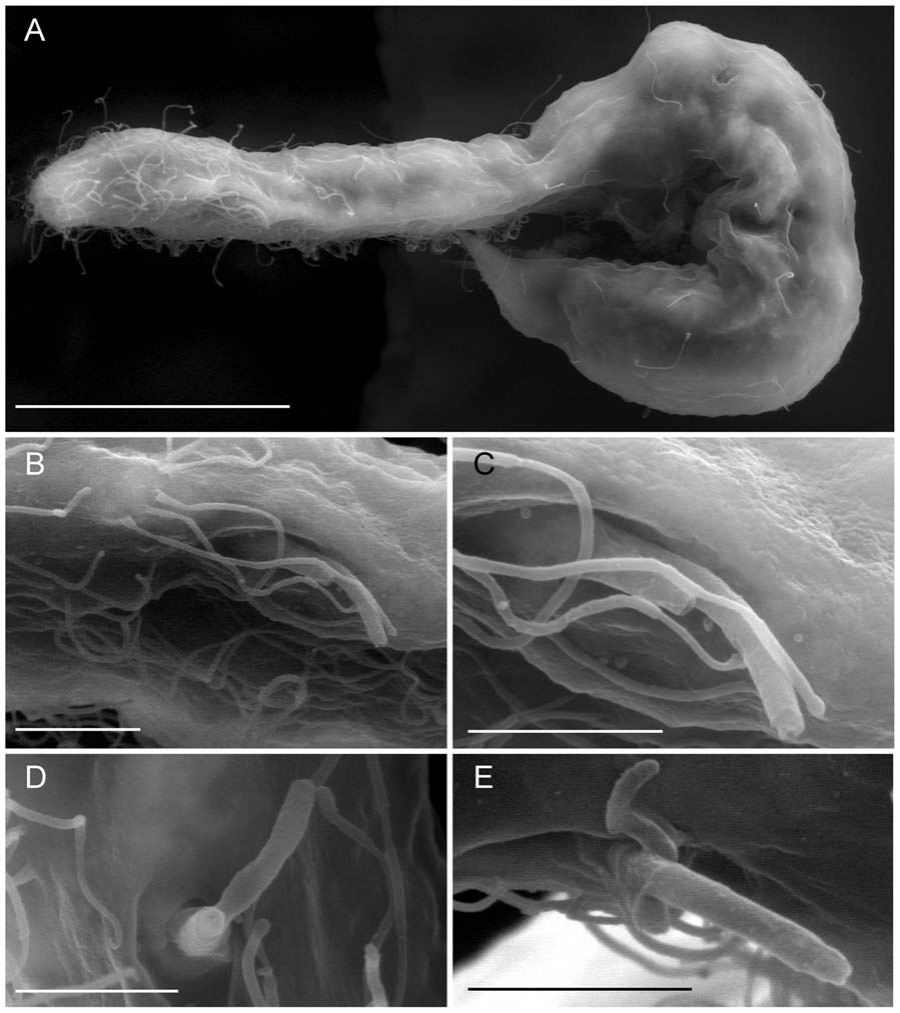
*Redudasys fornerise*. SEM photomicrographs. A, adult specimens, bent, lateral view; C–E, close up of the anterior adhesive tubes from various specimens. Scale bars, A, 50 µm, B–E, 5 µm.

*Redudasys fornerise* (Figures 1, 2) was found in sandy sediments collected on 12 February 2008 from the Represa do Broa on Rio do Lobo, located near the town of Itirapina, state of São Paulo, Brazil.

Ten sand-filled 0.5-L plastic jar samples, collected in several parts of the lake, were brought within 24 hr to the laboratory at the University of Saõ Paulo and analysed for gastrotrichs during a one-week period. To find gastrotrichs, subsamples were treated with 1% MgCl_2_-solution to anaesthetize the animals [5]. Specimens were localized under a Wild M8 stereomicroscope, transferred to a slide with a micropipette and studied alive. Three specimens were fixed in 10% borax neutralized formalin and stored for later SEM analysis; five additional specimens were fixed and kept in absolute ethanol for future DNA analysis. Other animals not used in this study were fixed and stored for ultrastructural investigations.

### Morphological analysis

Light microscopy: Eight living relaxed specimens were studied under Nomarski differential interference contrast (DIC) optics using a Zeiss Axioscop 2 Plus microscope. During observation, the specimens were measured using an ocular micrometer and photographed with a Nikon Coolpix 995 digital camera (3.34 Mpixel). In the morphological account, the positions of certain anatomical traits are given in percentage units (U) of total body length measured from anterior to posterior [6].

Scanning electron microscopy: For SEM, the formalin-fixed worms were rinsed in 0.1 M PBS, dehydrated through a graded ethanol series, critical point-dried using CO_2_, mounted on aluminium stubs, sputter coated with gold-palladium and observed with a Philips XL 30 microscope [7].

### Molecular analysis

**Figure 3:**
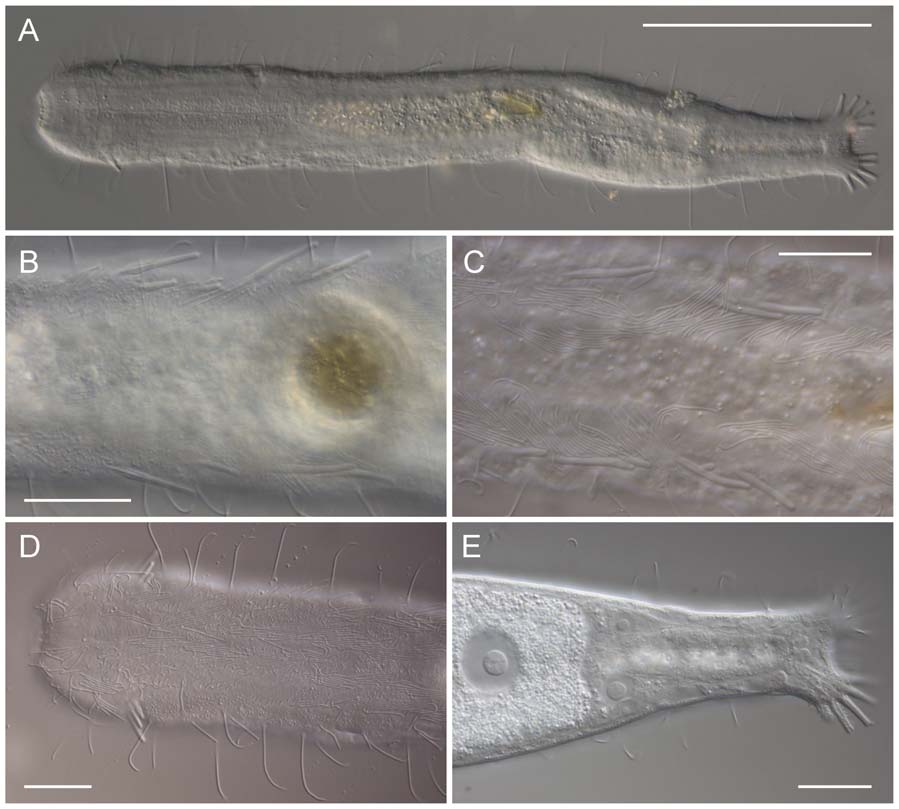
*Anandrodasys agadasys* ( = *Dactylopodola agadasys*) from the US Virgin Islands. A, habitus of a fully relaxed adult specimen; B, C adhesive tubes of the ventrolateral series of two different adult specimens; D, close-up of the anterior end of a fourth specimens, showing the arrangement of sensorial cilia and the insertion of the anterior adhesive tubes; E, posterior trunk region showing the female reproductive apparatus with eggs at different developing stages. DIC photomicrographs. Scale bars, A, 100 µm, B–E, 20 µm. Originally described from Australia, later the species has been reported from Panama, Red sea, Caribbean sea and Florida [6], [24]. According to Hummon [6] there are not morphological differences among populations. Morphology of the specimens from St John match that describe d by Hummon [6]; however, we noticed some variability in the number and arrangement of the ventrolateral adhesive tubes, as testified by Figures B and C.

Selection of taxa: To estimate the phylogenetic relationships of *R. fornerise* within the order Macrodasyida, we used the near complete 18 S rDNA genes sequences of 42 species (43 specimens) belonging to 23 genera within the eight currently recognized families (Tables 1, 2). A representative of the order Chaetonotida, *Xenotrichula intermedia* (Xenotrichulidae), was chosen as the out-group in the analyses. Most of the sequences were recently obtained by some of the authors [8], and together with a few more [9]–[12] were downloaded from GenBank (Table 2). Sequences belonging to the Brazilian worms and to the four additional species *Crasiella diplura* Clausen, 1968 (Planodasyidae), *Dactylopodola agadasys* Hochberg, 2003 (Dactylopodolidae, Figure 3), *Pleurodasys helgolandicus* Remane, 1927 (Cephalodasyidae) and *Xenodasys riedli* (Schoepfer-Sterrer, 1969) (Xenodasyidae) were obtained for the purpose of this study. The inclusion in the analysis of these new sequences is particularly important as representatives of the taxa involved share with *R. fornerise* a suite of important, potentially homologous morphological characteristics e.g. the arrangement of the adhesive tubes of the anterior series, the appearance of the posterior end, and/or clearly visible cross-striated longitudinal muscles. Specimens for the new sequences were found during a number of faunistic surveys headed by the senior author and conform to the latest morphological account provided for each species they represent; no special permission/permits were needed to collect these animals as gastrotrichs are microscopic, non-pathogenic organisms. Field study did not involve endangered species and sampling was carried out in public beaches. Soon after sampling, these gastrotrichs were extracted from the sandy substrata using a 7% MgCl_2_ solution [13], fixed in 95% Ethanol and stored at −20°C until further treatment. Full lists of specimens, together with sampling locations as well as geographic coordinates and GenBank accession numbers are presented in Tables 1 and 2.

**Table 1:** New sequenced gastrotrich taxa used in this study.

Taxon	Origin	Coordinates	accession
**Cephalodasyidae**			
*Pleurodasys helgolandicus*	Ibiza, Spain	39°03′07″N; 01°35′41″E	JN203486
**Dactylopodolidae**			
*Dactylopodola agadasys*	St. John Island, USA	18°19′11″N; 64°43′34″W	JN203487
**Planodasyidae**			
*Crasiella* sp.	Ilha Bela, Brazil	23°50′30″S; 45°24′14″W	JN203488
**Xenodasyidae**			
*Xenodasys riedli*	St. John Island, USA	18°19′11″N; 64°43′34″W	JN203490
***Incertae sedis***			
*Redudasys fornerise*	Represa do Broa, Brazil	22°11′10″S; 47°54′02″W	JN203489

Sampling locations together with their respective coordinates are given as well as GenBank accession number.

**Table 2:** Additional gastrotrich taxa used in this study.

Taxon	Origin	Reference	accession
**Cephalodasyidae**			
*Cephalodasys* sp	White Sea, Russia	[10]	AY963691
*Dolichodasys* sp.	San Isidoro, Italy	[9]	AM231778
*Megadasys* sp.	Grotta del Ciolo, Italy	[8]	JF357655
*Megadasys* sp. 1	Porto Cesareo, Italy	[8]	JF357656
*Mesodasys laticaudatus*	Albinia, Italy	[8]	JF357657
*Mesodasys littoralis*	Bou Ficha, Tunisia	[8]	JF357658
*Paradasys* sp.	Ionian sea, Italy	[9]	AM231781
**Dactylopodolidae**			
*Dactylopodola* cf. *baltica*	Ras Alard, Kuwait	[8]	JF357650
*Dactylopodola mesotyphle*	Punta Ala, Italy	[8]	JF357651
*Dactylopodola typhle*	Bou Ficha, Tunisia	[8]	JF357652
*Dactylopodola typhle*	Torre Civette, Italy	[8]	JF357653
**Lepidodasyidae**			
*Lepidodasys unicarenatus*	Pianosa, Italy	[8]	JF357665
**Macrodasyidae**			
*Macrodasys* sp. 1	Torre Civette, Italy	[8]	JF357654
*Macrodasys* sp. 2	Bohuslän, Sweden	[8]	JF357670
*Urodasys* sp.1	NA	[11]	AY218102
*Urodasys* sp.2	Florida, USA	[12]	DQ079912
**Thumastodermatidae**			
*Acanthodasys* sp. A	Capraia, Italy	[8]	JF357638
*Acanthodasys aculeatus*	Capraia, Italy	[8]	JF357639
*Diplodasys ankeli*	Meloria, Italy	[8]	JF357624
*Diplodasys meloriae*	Meloria, Italy	[8]	JF357640
*Oregodasys ocellatus*	Meloria, Italy	[8]	JF357642
*Oregodasys ruber*	Meloria, Italy	[8]	JF357625
*Oregodasys tentaculatus*	Meloria, Italy	[8]	JF357626
*Pseudostomella etrusca*	Albinia, Italy	[8]	JF357633
*Ptychostomella* sp. 1	Ilha Bela, Brazil	[8]	JF357643
*Ptychostomella tyrrhenica*	Albinia, Italy	[8]	JF357634
*Tetranchyroderma papii*	Sardegna, Italy	[8]	JF357637
*Tetranchyroderma esarabdophorum*	Mahdia, Tunisia	[8]	JF357627
*Tetranchyroderma hirtum*	Capraia, Italy	[8]	JF357628
*Tetranchyroderma thysanophorum*	Albinia, Italy	[8]	JF357630
*Thaumastoderma moebjergi*	Bohuslän, Sweden	[8]	JF357671
*Thaumastoderma ramuliferum*	Meloria, Italy	[8]	JF357631
**Turbanellidae**			
*Paraturbanella dohrni*	Punta Ala, Italy	[8]	JF357659
*Paraturbanella pallida*	Capraia, Italy	[8]	JF357660
*Paraturbanella teissieri*	Punta Ala, Italy	[8]	JF357661
*Turbanella bocqueti*	Tramore, Ireland	[8]	JF357662
*Turbanella cornuta*	Chioggia, Italy	[8]	JF357663
*Turbanella lutheri*	Torö, Sweden	[8]	JF357669
**Xenotrichulidae** *			
*Xenotrichula intermedia*	Mahdia, Tunisia	[8]	JF357664

*Order Chaetonotida.

Origin, reference and GenBank accession number are given. NA, not available.

### DNA extraction and amplification

DNA was extracted from single, whole specimens using the QIAamp DNA mini kit (QIAGEN), with columns from the QIAamp DNA micro kit (QIAGEN) according to the manufacturer's instructions. The extraction yielded two extracts of 20 and 40 µl respectively for each specimen; DNA from the first extract was used as template for the subsequent amplifications. Over 1700 bp of DNA was amplified using the 0.2 ml PuReTaq Ready-To-Go PCR beads (GE Healthcare). For amplification, 0.5 µl of each primer, 2 µl of DNA and 22 µl of purified water were assembled in the RTG-PCR tubes yielding a final volume of 25 µl. Primer sequences and PCR-programs are the same as in Todaro et al. [8]. Polymerase chain reactions were made in a Gene Amp PCR System 9700 (Applied Biosystems) or in a Biometra personal thermocycler. In some cases the PCR-product had to be purified with the QIAquick PCR Purification Kit (QIAGEN) according to the manufacturer's instructions. To remove excess nucleotide fragments EXO and SAP (Fermentas) were mixed in proportions 1∶4 and subsequently 5.5 µl EXOSAP added to all PCR-products. Sequence reactions were made according to the BigDye® Terminator v3.1 Sequencing Standard Kit (Applied Biosystems) following the manufacturer's instructions. An ABI3130XL Automated DNA sequencer (Applied Biosystems, Hitachi) was used to produce chromatograms. Purified PCR product from *D. agadasys* and *X. riedli* was sent for sequencing to Macrogen, Korea (www.macrogen.co.kr).

### Alignment and Phylogenetic analyses

New contigs were assembled using Staden v 1.6.0 [14]. The 44 sequences were aligned with ClustalX using the default parameters. The data set, which consisted of 1857 nucleotide characters, was subsequently converted into both interleaved nexus and fasta formatted files and analysed phylogenetically using three different approaches: i) Bayesian inference (MrBayes 3.1.2), [15], ii) Maximum Likelihood and iii) Maximum Parsimony (Mega 5) [16]. For the analysis carried out with MrBayes, we used the evolutionary model of nucleotide substitution GTR+G+I, favoured by both the AICc and the lnL criterion in MrModeltest v2.3 [17]. Two trials with four simultaneous chains were run for 6000000 generations; trees were sampled every 100^th^ generation after a burnin of 15000 generations. A 50% consensus tree was produced with TreeView [18]. For the ML analysis we used the K2+G+I model, which gained the best fit score under the AICc and lnL criteria in Mega 5. For both the ML and MP analyses, we selected the “use-all sites” data treatment option and set the phylogeny test to bootstrap with 1000 replication.

### Nomenclatural acts

The electronic version of this document does not represent a published work according to the International Code of Zoological Nomenclature (ICZN), and hence the nomenclatural acts contained in the electronic version are not available under that Code from the electronic edition. Therefore, a separate edition of this document was produced by a method that assures numerous identical and durable copies, and those copies were simultaneously obtainable (from the publication date noted on the first page of this article) for the purpose of providing a public and permanent scientific record, in accordance with Article 8.1 of the Code. The separate print-only edition is available on request from PLoS by sending a request to PLoS ONE, Public Library of Science, 1160 Battery Street, Suite 100, San Francisco, CA 94111, USA along with a check for $10 (to cover printing and postage) payable to “Public Library of Science”.

In addition, this published work and the nomenclatural acts it contains have been registered in ZooBank, the proposed online registration system for the ICZN. The ZooBank LSIDs (Life Science Identifiers) can be resolved and the associated information viewed through any standard web browser by appending the LSID to the prefix “http://zoobank.org/”. The LSID for this publication is: urn:lsid:zoobank.org:pub:0C88A867-EC44-4481-B35A-A3D49D268EDC

Printed copies will be deposited at the following libraries:

Swedish Museum of Natural History (Stockholm, Sweden);

The Natural History Museum (London, United Kingdom);

Smithsonian National Museum of Natural History (Washington D.C., USA);

Queensland Museum (Brisbane, Australia);

Museo de Zoologia, Universitade de São Paulo (São Paulo, Brazil).

## Results

### Morphology of Redudasys fornerise

Sexually mature specimens range 390–405 µm in total body length; pharynx length 135–140 µm; pharyngeo-intestinal junction (PhJIn) at U35–U36 (Figure 1). Body flattened ventrally, vaulted dorsally, comprised of bluntly tapered head bearing evident sensorial cilia; neck constriction extended but slight; trunk slender, slightly broadened at mid trunk then narrowing at the base of the two lobed caudum that indents at U94; each caudal lobe has two diverging adhesive tubes (Figure 1A,B). Widths of head/neck/trunk/caudal base are as follows: 58/45/72/27 µm at U10.5/U26/U68/U93, respectively. The body surface appears smooth and transparent, without cuticular formation such as spines or scales (Figures 1A,B, 2A); epidermal glands absent; protonephridia present, 3 (or 4?) per side, located just past the PhJIn (U36.6), at mid- and in the hindgut region at U63 and U71, respectively; the more posterior nephridial structure on each side appears larger and structurally more complex than the anterior structure, and may in fact be made each of two adjacent, yet independent filtering units. Longitudinal muscles clearly visible and cross-striated.

Adhesive tubes: TbA 2 per side, one shorter (9 µm in length) than the other (13 µm in length) (Figure 1A, C). SEM shows that the tubes of each group are borne from a common base (3 µm long) emerging from a ventrolateral furrow protected on top by a shallow cuticular roofing; the common base emerges with a slightly oblique orientation so that the more ventrally positioned smaller adhesive tube appears slightly anterior compared to the longer tube (Figures 1D, 2B–E). TbP 2 per each caudal lobe (L 13–17 µm), shortest medially on each lobe (Figure 1A,B). TbL, TbD and TbV are absent.

Ciliation: Sensory hairs (8–25 µm in length) are abundant on the anterior end, to U17, but become scarce on the rest of the body (Figures 1A,B, 2A). At the frontal end, at least 10 short and stiff hairs encircle the mouth, while an additional 20–40 longer hairs are inserted, sometimes in groups of two or three, on the top and lateral sides of the head (Figure 1D). Body hairs are arranged in two lateral (U20–U93) and two dorsolateral (U40–U91) columns containing each 5–6 equally spaced groups. Sensorial hairs of the ventrolateral series are shorter (10 µm) than groups of the lateral series (18 µm long). Locomotor cilia are distributed ventrally in separate ciliary fields of unequal size; fields are paired posterior to the mouth and along the pharyngeal region (U04–U36), then become unpaired patches along the median line of the trunk region (U50–U39).

Digestive tract: Mouth is terminal, slightly inclined ventrally, and of small breadth (5–6 µm in diameter); the buccal cavity is 17 µm long, not lined with evident cuticle but supported by a strong musculature. Pharynx broadest in the buccal region, its breadth following the body contours in the head and neck region, with evident pharyngeal pores at base, that open ventrolaterally at U31. Foregut broad, midgut narrowing, hindgut broadening slightly before the anus, which occurs ventrally at U90.

Reproductive tract: Probably parthenogenetic; male system not seen; ovaries paired in hindgut region, with oocytes (3–4 or more) per side behind the predominant ovum (56×28 µm), which develops medially forward toward the midgut; caudal and frontal organs not seen.

Ecology: Occasional in frequency of occurrence (10–30% of samples), dominant in sample where found; shallow sublittoral (0.7 m water depth) in medium (M = 0.379 mm), well sorted (SD = 0.69 mm) siliceous sand with some detritus.

### Phylogenetic analysis

**Figure 4:**
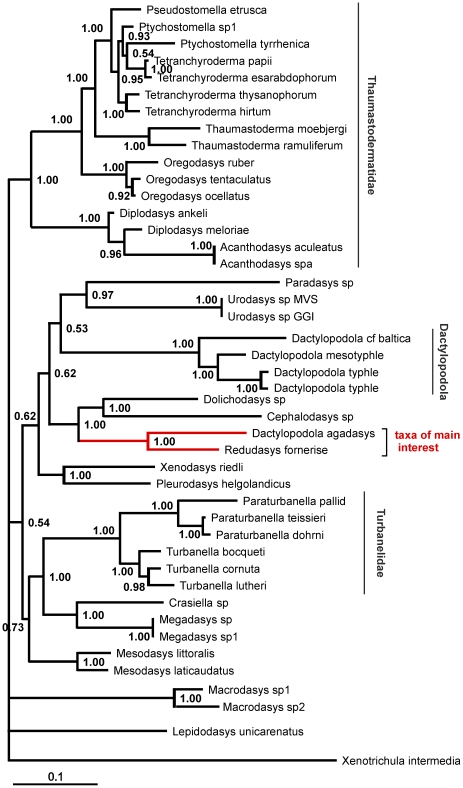
Phylogenetic relationships of 43 Gastrotricha Macrodasyida inferred from Bayesian analysis of 18 S rDNA. The outgroup is represented by *Xenotrichula intermedia* (Chaetonotida, Xenotrichulidae). Number at nodes represents posterior probabilities.

The final dataset included 1857 alignable positions, 969 of which are constant and 707 parsimony-informative. The three phylogenetic analyses, carried out with ML, MP and Bayesian approaches, yielded topologies highly congruent with each other, with most of the many groups that are in common bearing high nodal support: i.e. bootstrap and Bayesian posterior probability values ≥75 and 98% respectively (Figures 4–6). Among the robustly supported groups is the novel alliance between *Redudasys fornerise* and *Dactylopodola agadasys* and the currently recognized sub-groupings within the densely sampled families Thaumastodermatidae and Turbanellidae.

**Figure 5 pone-0031740-g005:**
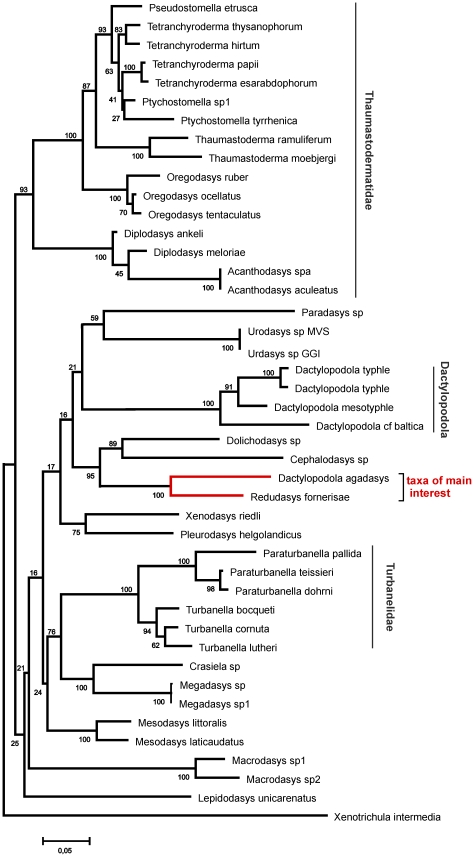
Phylogenetic relationships of 43 Gastrotricha Macrodasyida inferred from Maximum Likelihood analsysis of 18 S rDNA. The outgroup is represented by *Xenotrichula intermedia* (Chaetonotida, Xenotrichulidae). The tree is drawn to scale, with branch lengths measured in the number of substitutions per site. Number at nodes represents bootstrap values (1000 replicates).

**Figure 6:**
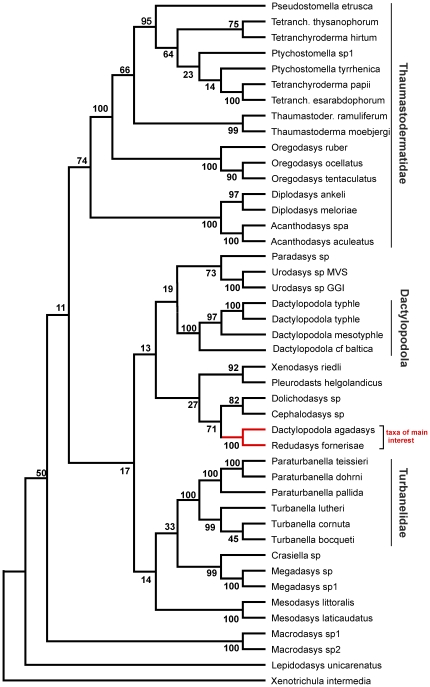
Phylogenetic relationships of 43 Gastrotricha Macrodasyida inferred from Maximum Parsimony analysis of 18 S rDNA. The outgroup is represented by *Xenotrichula intermedia* (Chaetonotida, Xenotrichulidae). Tree #1 out of 3 most parsimonious trees (length = 3746) is shown. The consistency index is (0.382720), the retention index is (0.608797), and the composite index is 0.254573 (0.232999) for all sites and parsimony-informative sites (in parentheses). Number at nodes represents bootstrap values (1000 replicates).

By contrast, Macrodasyidae and Cephalodasyidae never appear as monophyletic due to the scattering along the evolutionary tree of their respective species and/or the “unorthodox” alliances between members of different families. In this regard, there is the noteworthy recovery of two strongly supported clades made up of *Pleurodasys helgolandicus* (Cephalodasyidae)+*Xenodasys riedli* (Xenodasyidae) and especially *Megadasys* spp (Cephalodasysidae)+*Crasiella* sp. (Planodasyidae).

Genera represented by two or more species were in general recovered as monophyletic in our analyses with the notable exception of *Dactylopodola*. Of the four species (5 terminals) included in our study, three species (4 terminals) formed a distinct clade with high bootstrap support, while *D. agadasys* formed a separate grouping with *R. fornerise* (Figures 4–6).

## Discussion

*Redudasys fornerise* was originally described based on observations carried out using bright field and phase contrast microscopy [2]; both of these techniques are less powerful than the microscopical methods such as DIC and SEM that are currently used for surveying gastrotrich anatomy [13]. Still, the original description of *R. fornerise* appears to be generally correct when compared with data obtained in our investigation; differences pertain to the insertion and arrangement of the anterior tubes, location and perhaps number of protonephridia, and to the different number and distribution of dorsal and lateral sensorial bristles; this latter trait is however somewhat variable among the animals we have observed. Most important, our survey confirms the absence of the male reproductive organs and gametes in mature specimens of *R. fornerise*; consequently, from a reproductive point of view, these animals can be reasonably considered parthenogenetic.

Within Macrodasyida, parthenogenesis is a very rare phenomenon. Reliable records (i.e. same information from different authors) include two other species only i.e., *Urodasys viviparus* (Macrodasyidae) and *Dactylopodola agadasys* (Dactylopodolidae). While it is hard to find morphological similarities between *R. fornerise* and *U. viviparus* (or any other *Urodasys* species) several common traits emerge that may unite *R. fornerise* and *D. agadasys* (Figure 3). Beside the similar size and general appearance, both species have anterior adhesive tubes distributed in two groups, a bilobed caudum, three pairs of protonephridia, and clearly visible striated longitudinal muscles. Number and arrangement of adhesive tubes of the anterior and posterior series are also quite similar across the two taxa.

Phylogenetic analyses based on 18 S sequence data support the grouping of *R. fornerise* and *D. agadasys*, and therefore point to the homologous nature of the morphological similarities noted above. On the other hand, these same analyses clearly separate *D. agadasys* from the other four *Dactylopodola* species studied indicating that morphological traits used to allocate *D. agadasys* to its current genus (e.g. arrangement of anterior adhesive tubes, bilobed caudam, striated longitudinal muscles etc) [19], may in fact be considered at best as plesiomorphies (see Figures 4–6). If this turns out to be true, it will be evident that the taxonomic importance given to some traits at that time was inappropriate and has led to the existing confusion.

Within Macrodasyida, statistically supported phylogenetic hypotheses resulting from analysis of the 18 S rRNA gene have so far proved to be robust and very likely i.e.: i) similar topologies are obtained by analyzing data sets of concatenated sequences of different genes [8]; and ii) topologies are congruent with evolutionary hypotheses obtained by analyzing morphological traits, as testified by the recovering as monophyletic of most of the genera and of the morphologically homogeneous families Turbanellidae and Thaumastodermatidae [8], [9], [20].

Previously, in assessing the “unorthodox” position of *D. agadasys* recovered in our analysis, we have pointed out that where contrasts exist between morphological and molecular scenarios, a reasonable re-evaluation of the morphological evidence may call off the hypothetical differences. Within this framework, a close phylogenetic relationship between *R. fornerise* and *D. agadasys* as hypothesized by our molecular analyses appears highly realistic. Incidentally, this provides support to the latent doubts of Hochberg [19] who in describing and naming *D. agadasys* pointed out that the Australian species was indeed quite different from any other known species of *Dactylopodola*, notwithstanding the number of shared morphological similarities. In fact, clear differences separate ‘genuine’ species of *Dactylopodola* from *D. agadasys: i*) body tenpin shaped *vs* vermiform, *ii*) head distinct from the trunk by a well defined neck constriction *vs* head weakly marked and absence of a true neck constriction, iii) pharynx generally short, confined for most part within the head and neck regions *vs* pharynx comparatively longer and extending well past the neck region; *iiii*) reproductive system including both male and female organs (i.e. hermaphroditic) *vs* presence of the female gonad only (i.e. parthenogenetic).

Our findings have taxonomic and ecological consequences. From a taxonomic perspective, it is now necessary to reclassify the species originally described from Australia since it is no longer related to other species of *Dactylopodola*; we propose to erect the new genus, *Anandrodasys* gen. nov., to contain all members of the previously described *D. agadasys*. Further, we propose the name Redudasyidae fam. nov. to include both the genera *Redudasys* and *Anandrodasys;* the diagnoses of these new taxa are provided below.

From an ecological perspective, the derived position of *Redudasys*, which is well nested within the Macrodasyida clade (Figures 4–6), unequivocally demonstrates that the colonization of freshwater habitats by macrodasyidan Gastrotricha has taken place independently from a similar colonization by chaetonotidan Gastrotricha. This double- freshwater invasion hypothesis was recently challenged by Kieneke et al. [21] who, on the basis of a cladistic analysis of morphological characters, found *Redudasys* allied with *Marinellina* in a clade basal to the freshwater Chaetonotida Paucitubulatina, thus insinuating that the invasion of freshwater systems by Gastrotricha happened only once. It should be highlighted however that the topology obtained by Kieneke et al. [21] was plagued by low bootstrap support at most nodes, including this specific one, leaving little confidence in any proposed hypothesis.

Phylogenetic trees resulting from the current analyses show several other well supported clades, some of which include taxa belonging to different families e.g. *Pleurodasys helgolandicus* (Cephalodasyidae)+*Xenodasys riedli* (Xenodasyidae), *Megadasys* spp (Cephalodasysidae)+*Crasiella* sp. (Planodasyidae) etc. At the same time, analyses fail to recover as monophyletic some of the currently well-recognized high ranking taxa (e.g. Macrodasyidae).

While we believe some of these novel phylogenetic hypotheses to be suggestive and potentially interesting, a thoughtful discussion about them falls beyond the scope of this study. Instead, we caution that phylogenetic research into Gastrotricha still remains relatively young, and that new and appealing hypotheses can be quickly dismissed once taxon sampling improves for all respective families and genera [8], [22].

Diagnoses

**Redudasyidae** fam. nov.

Macrodasyidans about 400 µm in total length, with weakly marked head bearing several sensorial cilia but without tentacles or ocelli. Lateral trunk margins even, without indentations or protrusions. Posterior end two lobed, without a peduncle. Cuticular covering smooth, without scales or spines. Adhesive apparatus consisting of anterior and posterior tubes; ventrolateral tubes may also be present (*Anandrodasys*). TbA, distributed in two symmetrical groups made each of two-three tubes of unequal length; tubes of each group borne from a common base emerging from a ventrolateral furrow (*Redudasys*) or insert in parallel, protruding obliquely to the rear (*Anandrodasys*). TbP, 4–12 in total, distributed symmetrically at the end of the two caudal lobes. Ventrolateral tube 5–6 per side, along the anterior intestinal region. Lateral and dorsal tubes absent. Longitudinal muscles visibly cross-striated. Protonephridia present, three-four per side. Ventral ciliation arranged in a unified field beneath the head that splits into a pair of longitudinal bands along the neck and trunk region and forms an isolated patch lying medially behind the anus (*Anandrodasys*) or as a reminiscence of this arrangement (*Redudasys*). Mouth, terminal or slightly subterminal; buccal cavity inconspicuous, not lined with evident cuticle. Pharynx bearing pores at base, opening ventrolaterally. Intestine straight; anus ventral. Parthenogenetic; ovaries paired in hindgut region, with oocytes behind the predominant ovum; male apparatus unknown; frontal and caudal organs unknown. Interstitial, marine or freshwater.

Type genus: *Redudasys* Kisielewski, 1987; other genera: *Anandrodasys* gen. nov.

Genus ***Redudasys*** (emended)

Macrodasyidan about 400 µm in total length, with weakly demarcated head bearing several sensorial cilia but without tentacles or ocelli (*Redudasys fornerise*). Lateral trunk margins even, without indentations or protrusions. Posterior end two lobed, without a peduncle. Cuticular covering smooth, without scales or spines. Adhesive apparatus consisting of anterior and posterior tubes. TbA, distributed in two symmetrical groups made each of two tubes of unequal length; tubes of each group are borne from a common base emerging from a ventrolateral furrow (*Redudasys fornerise*). TbP, four in total, distributed symmetrically at the end of the two caudal lobes, medial tubes shorter than the others. Lateral and dorsal tubes absent. Longitudinal muscles visibly cross-striated. Protonephridia present, three per side. Ventral ciliation distributed in groups arranged in paired longitudinal series along the anterior part of body and unpaired median ones along the posterior trunk region. Mouth, terminal or slightly subterminal; buccal cavity inconspicuous, lined with thin cuticle. Pharynx bearing pores at base, opening ventrolaterally. Intestine straight, narrows fore to aft; anus ventral. Parthenogenetic; ovaries paired in hindgut region, with oocytes behind the predominant ovum; male apparatus absent; frontal and caudal organs absent. Freshwater, interstitial in medium siliceous sand. Thus far reported from Brazil only. Type species: *Redudasys fornerise* Kisielewski, 1987 (*sensu* Todaro et al., this publication). Other species: the taxonomic status of *Redudasys* sp. reported by Garrafoni et al. [23] has to be assessed.

***Anandrodasys*** gen. nov.

urn:lsid:zoobank.org:act:BBCA9AF9-A6C4-4F69-8C14-29D050565700

Macrodasyidan less than 400 µm in total length, with weakly demarcated head bearing several sensorial cilia but without tentacles or ocelli. Lateral trunk margins even, without indentations or protrusions. Posterior end two lobed, without a peduncle. Cuticular covering smooth, without scales or spines. Adhesive apparatus consisting of anterior, ventrolateral and posterior tubes. TbA, distributed in two symmetrical groups made each of three tubes of unequal length; tubes of each group insert in parallel, protruding obliquely to the rear, longest lateral. TbVL, 5–6 per side, along the anterior intestinal region. TbP, 6 per caudal lobe, longest medially on each lobe. Dorsal tubes absent. Longitudinal muscles visibly cross-striated. Protonephridia present, three per side. Ventral ciliation arranged in a unified field beneath the head, into a pair of longitudinal bands along the neck and trunk region, and in an isolated patch lying medially behind the anus. Mouth terminal, buccal cavity goblet-shaped; pharynx width follows the head/neck contours, with inconspicuous basal pores that open well behind the neck constriction; intestine straight, narrows fore to aft, anus ventral. Parthenogenetic; ovaries paired in hindgut region, with oocytes on both sides behind the predominant ovum; male system absent; caudal and frontal organs absent. Marine, interstitial in medium siliceous and calcareous sand. Thus far reported from Australia, Red and Caribbean Seas, Panama and Florida. Type species: *Anandrodasys agadasys* (Hochberg, 2003) (*sensu* Hummon [6] = *Dactylopodola agadasys* Hochberg, 2003). Other species: Disjunct populations from Australia, Red Sea, Caribbean sea, Panama and Florida, have so far been affiliated to the original species based on homogeneity of the morphological traits [6], [24].

Etymology: *- Anandrodasys* (*Anandros* Gr = without male and *dasys* Gr, hairy) the first word alludes to the parthenogenetic nature of these animals while the second appears in the name of most gastrotrich genera and alludes to their dense ciliation.

## References

[1] Ruttner-Kolisko A (1955). *Rheomorpha neiswestnovae* und *Marinellina flagellata*, zwei phylogenetische interessante Wurmtypen aus dem Süsswasserpsammon.. Österr Zool Z.

[2] Kisielewski J (1987). Two new interesting genera of Gastrotricha (Macrodasyida and Chaetonotida) from the Brazilian freshwater psammon.. Hydrobiologia.

[3] Hummon WD, Todaro MA (2010). Analytic taxonomy and notes on marine, brackish-water and estuarine Gastrotricha.. Zootaxa.

[4] Hummon WD (2010). Global database for marine Gastrotricha (Taxonomic, Geographic, Bibliographic, and Video).. http://hummon-nas.biosci.ohiou.edu.

[5] Kånneby T, Todaro MA, Jondelius U (2009). One new species and records of *Ichthydium* Ehrenberg, 1830 (Gastrotricha: Chaetonotida) from Sweden with a key to the genus.. Zootaxa.

[6] Hummon WD (2011). Marine Gastrotricha of the Near East: 1. Fourteen new species of Macrodasyida and a redescription of *Dactylopodola agadasys* Hochberg, 2003.. Zookeys.

[7] Dal Zotto M, Ghiviriga S, Todaro MA (2010). A new *Tetranchyroderma* (Gastrotricha, Thaumastodermatidae) with triancres from the Mediterranean Sea.. Meiofauna Mar.

[8] Todaro MA, Kanneby T, Dal Zotto M, Jondelius U (2011). Phylogeny of Thaumastodermatidae (Gastrotricha: Macrodasyida) inferred from nuclear and mitochondrial sequence data.. PLoS ONE.

[9] Todaro MA, Littlewood DTJ, Balsamo M, Herniou EA, Cassanelli S (2003). The interrelationships of the Gastrotricha using nuclear small rRNA subunit sequence data, with an interpretation based on morphology.. Zool Anz.

[10] Petrov NB, Pegova AN, Manylov OG, Vladychenskaya NS, Mugue NS (2007). Molecular phylogeny of gastrotricha on the basis of a comparison of the 18 S rRNA genes: Rejection of the hypothesis of a relationship between Gastrotricha and Nematoda.. Mol Biol.

[11] Giribet G, Sorensen MV, Funch P, Kristensen RM, Sterrer W (2004). Investigations into the phylogenetic position of Micrognathozoa using four molecular loci.. Cladistics.

[12] Sørensen MV, Sterrer W, Giribet G (2006). Gnathostomulid phylogeny inferred from a combined approach of four molecular loci and morphology.. Cladistics.

[13] Todaro MA, Hummon WD (2008). An overview and a dichotomous key to genera of the phylum Gastrotricha.. Meiofauna Mar.

[14] Staden R (1996). The Staden sequence analysis package.. Mol Biotechnol.

[15] Ronquist F, Huelsenbeck JP (2003). MRBAYES 3: Bayesian phylogenetic inference under mixed models.. Bioinformatics.

[16] Tamura K, Dudley J, Nei M, Kumar S (2007). MEGA4: Molecular Evolutionary Genetics Analysis (MEGA) software version 4.0.. Mol Biol Evol.

[17] Nylander JA (2004). MrModeltest v2..

[18] Page RDM (1996). TREEVIEW: An application to display phylogenetic trees on personal computers.. Comput Appl Biosci.

[19] Hochberg R (2003). Two new species of *Dactylopodola* (Gastrotricha, Macrodasyida) from islands off the Queensland coast, Australia.. Meiofauna Mar.

[20] Todaro MA, Telford MJ, Lockyer AE, Littlewood DTJ (2006). Interrelationships of the Gastrotricha and their place among the Metazoa inferred from 18 S rRNA genes.. Zool Scr.

[21] Kieneke A, Riemann O, Ahlrichs WH (2008). Novel implications for the basal internal relationships of Gastrotricha revealed by an analysis of morphological characters.. Zool Scr.

[22] Plazzi F, Ferrucci RR, Passamonti M (2010). Phylogenetic representativeness: a new method for evaluating taxon sampling in evolutionary studies.. BMC Bioinformatics.

[23] Garraffoni ARS, Araujo TQ, Lourenco AP, Balsamo M (2010). New data on freshwater psammic Gastrotricha from Brazil.. Zookeys.

[24] Hochberg R (2008). Gastrotricha of Bocas del Toro, Panama: A Preliminary Report.. Meiofauna Mar.

